# Editorial: Cell Communication in Vascular Biology, Volume II

**DOI:** 10.3389/fphys.2022.903056

**Published:** 2022-05-25

**Authors:** Mauricio P. Boric, Xavier F. Figueroa

**Affiliations:** Departamento de Fisiología, Facultad de Ciencias Biológicas, Pontificia Universidad Católica de Chile, Santiago, Chile

**Keywords:** endothelial cells, blood cells, vascular remodeling, endothelial dysfunction, lymphatic permeability, platelet miRNA, endothelial repair, endothelial ion channels

Communication is fundamental to integrate individual functions into complex systems, whether it be in communities, organisms, or cellular related interactions. Consistent with this, communication has provided the bases for the progress of civilizations as well as for the increasing complexity observed through the evolutionary process (Hornig, 1966; Torday and Rehan, 2009; State et al., 2015; Grouchy et al., 2016; Garcia et al., 2020). In higher vertebrates for instance, the functional coupling of the different cell types of the distinct organs and systems depends on a myriad of signaling mechanisms, which is reflected in the cardiovascular function by the harmonic integration of the vascular network with the surrounding tissues. Intercellular communication plays a central role in the function of the vascular system, since blood vessels possess a sophisticated architecture consisting of a special organization of distinct cell types, and then, control of vascular function depends on timely, fine intercellular communications across the vessel wall - mainly among endothelial cells, smooth muscle cells and perivascular nerves (Figueroa and Duling, 2009; Gaete et al., 2014; Gödecke and Haendeler, 2017; Majesky, 2018). Moreover, vessel wall cells must also work in coordination with cells that circulate in the blood stream such as red blood cells, platelets, and leucocytes, but, in addition, with parenchymal cells, other organs and systems to keep homeostasis in different physiological conditions (Gödecke and Haendeler, 2017; Krüger-Genge et al., 2019). Therefore, control of vascular function depends on different communication mechanisms between diverse cell types that are not always in direct contact with each other. The purpose of this Research Topic was to highlight the importance and diversity of cell communication in vascular biology, which could be appreciated in the first volume, but also in this second article collection (volume II). The volume II of this Research Topic comprises 6 articles in total: 2 Review articles, 1 Mini-Review article and 2 original contributions.

The circulatory system is a complex network in which the arterial and venous circulations are directly connected through the capillaries. Coordination of the complementary work of the arterial and venous systems is essential for the long-term function of the cardiovascular system (Hester and Hammer, 2002; Aitsebaomo et al., 2008; dela Paz and D’Amore, 2009); however, in addition to these, the homeostasis of the vascular network also relies on the lymphatic system (Breslin et al., 2018). During circulation, blood leaks plasma components from capillaries and postcapillary venules and lymphatic vessels are critical to return to the central circulation the capillary ultrafiltrate and extravasated proteins preventing a gradual reduction in plasma volume, with the consequent formation of edema (Breslin et al., 2018; Oliver et al., 2020). In addition, lymphatics are also important in the lipid absorption from the digestive tract and in the immunological responses (Oliver et al., 2020). Interestingly, in contrast to what was initially thought, lymphatic vasculature is also permeable to fluids and macromolecules such as albumin, but the physiological relevance of the lymphatic endothelial permeability and the mechanisms involved in this process are controversial (Breslin et al., 2018; Norden and Kume, 2021; Si, 2021). In this context, it is relevant to note that the adherens junction protein, VE-cadherin, and transendothelial vesicle transport were found to regulate different cellular pathways of the endothelial barrier function in blood vessels, which led to evaluate the potential involvement of these mechanisms in lymphatic vasculature (Breslin et al., 2018; Norden and Kume, 2021). Although previous studies contributed important molecular and morphological information, they failed to demonstrate the functional relevance of these mechanisms in the control of lymphatic endothelium permeability in intact lymphatic vessel, which is directly addressed in the article of (Jannaway and Scallan).

In addition of the complementary work of arterial, venous and lymphatic vasculature, vascular function also relies on the communication among cells circulating in the blood stream in dynamic coordination with the changes in the vessel wall observed at vascular microenvironments’ level (Augustin and Koh, 2017). In this context, platelets play important roles in many physiological and pathological processes (Rendu and Brohard-Bohn, 2001). Although platelets are typically thought to be quite simple, they are actually very complex. These circulating cells are known to be essential for the control of hemostasis and thrombosis; nevertheless, they also participate in the regulation of inflammation, wound healing, and angiogenesis by the release of diverse factors, such as proteins, chemokines, growth factors as well as proangiogenic and antiangiogenic signals (Rendu and Brohard-Bohn, 2001; Sharun et al., 2021). It should be noted, however, that platelet-mediated signaling have also been found to be involved in several aspects of cancer biology, including cancer growth and metastasis (Liu et al., 2021). Interestingly, in addition to possess a broad signaling repertoire, platelets can rapidly adapt to different vascular microenvironments through genetic modification mechanisms, such as intercellular transfer of messenger RNAs (mRNA) and microRNAs (miRNA) (Clancy and Freedman, 2014; Rondina and Weyrich, 2015). This is a novel aspect of platelet biology and all the potential delivery mechanisms that may be involved in the horizontal transfer of miRNA by platelets are discussed in detail in this Research Topic by Mussbacher et al. (Mussbacher et al.).

Homeostasis of each cell of the organism relies on the fine regulation of blood flow supply according to the changes in the metabolic demand of the tissues. Therefore, variations in cell activity must be paralleled by coordinated modifications in the diameter of resistance arteries controlling the distribution of local blood flow to the tissues (Segal et al., 2000; Segal, 2005). The magnitude of vessel diameter depends on the degree of constriction of smooth muscle cells in the vessel wall (i.e., vasomotor tone), which, in turn, is determined by the intracellular Ca^2+^ concentration ([Ca^2+^]_i_) and Ca^2+^ sensitivity of the contractile apparatus (Brekke et al., 2006; Earley and Nelson, 2006). The level of smooth muscle [Ca^2+^]_i_ is mainly dependent on the Ca^2+^ influx through L-type, voltage-dependent Ca^2+^ channels; then, the ion channels that control the membrane potential play a central role in the tonic regulation of vasomotor tone (Gollasch and Nelson, 1997). Thereby, on the first hand, smooth muscle cells depolarization produces a Ca^2+^ influx that leads to vasoconstriction and, in contrast, hyperpolarization results in a decrease in [Ca^2+^]_i_ that leads to vasodilation. Interestingly, on the other hand, an increase of [Ca^2+^]_i_ in endothelial cells triggers the activation of vasodilator signals, such as nitric oxide (NO) and a signaling pathway that is initiated by the opening of Ca^2+^-activated K^+^ channels (K_Ca_) of small (SK_Ca_) and intermediate (IK_Ca_) conductance in endothelial cells and leads to smooth muscle cell hyperpolarization (Lillo et al., 2005; Figueroa and Duling, 2009; Nilius and Droogmans, 2017). Therefore, the ion channels that are involved in the control of endothelial cell membrane potential and Ca^2+^ influx are critical signaling elements in the regulation of vascular function, as described by (Jackson W). However, ion channels can also be involved in the progress of the endothelial dysfunction observed in cardiovascular-related pathophysiological conditions, such as hypertension, obesity, diabetes mellitus and ageing; as explained by Goto and Kitazono (Goto and Kitazano), who highlight the participation of endothelial transient receptor potential vanilloid 4 (TRPV4) ion channel in the endothelial dysfunction associated with cardiovascular disease risk factors.

The arterial system is a complex network in which, at least, two functionally different vascular segments that must work in concert can be recognized: the conduit and resistance arteries (Davis et al., 1986; Izzo and Mitchell, 2007). Although the cellular composition of these two arterial segments is similar, the architecture of their vessel wall is designed to perform different functions, and consistent with this, the structure, cellular organization, and intercellular communication mechanisms varied along the arterial arborization and among the vascular territories (Aird, 2007; Augustin and Koh, 2017). In addition, the wall of arteries can be adapted or remodeled according to the changing functional requirements observed during the prevalence of different physiological conditions, as demonstrated by Villar-Fincheira et al. in the case of high training athletes in which soluble interleukin-6 receptor was found to regulate the interleukin-6-dependent vascular remodeling (Villar-Fincheira et al.). Likewise, the integrity of the endothelial layer lining the luminal surface of the vessels must be preserved to keep a proper vascular function (Karshovska et al., 2007; Tesfamariam, 2016) and, interestingly, in this article collection, the novel study of Kang et al. shows that zyxin plays a central role in the endothelial repair initiated by a cAMP-mediated signaling pathway after vascular injury (Kang et al.).

We understand that these fine articles will provide the reader with an appealing Volume II of the Cell Communication in Vascular Biology.
